# Bat activity correlated with migratory insect bioflows in the Pyrenees

**DOI:** 10.1098/rsos.230151

**Published:** 2023-08-16

**Authors:** Will L. Hawkes, Kelsey Davies, Scarlett Weston, Kelly Moyes, Jason W. Chapman, Karl R. Wotton

**Affiliations:** ^1^ Centre for Ecology and Conservation, University of Exeter, Cornwall Campus, Penryn, UK; ^2^ Environment and Sustainability Institute, University of Exeter, Cornwall Campus, Penryn, UK; ^3^ Department of Entomology, Nanjing Agricultural University, Nanjing, People's Republic of China; ^4^ Swiss Ornithological Institute, Sempach, Switzerland

**Keywords:** *Helicoverpa armigera*, ecosystem services, predator–prey interactions, nocturnal insect migration, Chiroptera, co-migrants

## Abstract

High altitude mountain passes in the Pyrenees are known to be important migratory hotspots for autumn migrating insects originating from large swathes of northern Europe. In the Pyrenees, prior research has focused on diurnal migratory insects. In this study, we investigate the nocturnal component of the migratory assemblage and ask if this transient food source is also used by bat species. Three seasons of insect trapping revealed 66 species of four different orders, 90% of which were Noctuid moths, including the destructive pest *Helicoverpa armigera*, otherwise known as the cotton bollworm. Acoustic bat detectors revealed that high activity of *Nyctalus* spp. and *Tadarida teniotis* bats were closely synchronized with the arrival of the migratory moths, suggesting this food source is important for both resident and migratory bats to build or maintain energy reserves. Bats of the *Nyctalus* spp*.* are likely migrating through the study site using fly-and-forage strategies or stopping over in the area, while resident *T. teniotis* may be exploiting the abundant food source to build fat stores for hibernation. This study shows that nocturnal migratory insects are abundant in the Pyrenees during autumn and interact during migration, not only with their co-migrant bats but also with resident bat species.

## 1. Introduction

Nocturnal migrants include many species of bird along with more commonly recognized nocturnal organisms such as moths and bats (e.g. [1,2]). This remarkable life-history trait sees individuals travel long distances to exploit seasonal resources and improve their reproductive success [3]. Migratory species' routes often converge with different taxa in certain locations leading to migratory bioflows of many species that are termed co-migrants. This co-migration can be caused by a variety of factors including using similar seasonal cues (such as temperature or day length change) to instigate their journeys, seasonal patterns of resources, specific currents of wind or water, or by geographical barriers [4]. The interactions between these co-migrants occur at a time in an organism's annual cycle when the energetic demands placed on their bodies are great, potentially increasing the impacts of the interactions and affecting the cost of migration [5,6].

Bats are known to interact with nocturnal insects and other taxa while on migration in the form of predation. In Europe, insectivorous Nathusius's pipistrelles (*Pipistrellus nathusii*) are thought to feed while actively migrating [7]. This is termed as a ‘fly and forage' strategy, although it is worth noting that the insects consumed are not known migrants [7,8]. Greater noctule bats (*Nyctalus lasiopterus*) are thought to have tightly comparable migration routes to those of nocturnally migrating passerines, as the bats will feed on their co-migrating birds during this period [9]. Some species of migratory bats have been known to exploit the movements of nocturnally migrating animals outside of the bats' own migratory period to rear young or to prepare for migration. In North America, *T. brasiliensis* bats are known to exploit the arrival of migratory moths to provide the nutrients they need to rear their pups [10–12]. Similarly, the *Tadarida* species found in Europe (*T. teniotis*) although sedentary, is thought to rely on migratory insects in order to provide the nutrients needed for their autumn-born pups to develop and to stockpile enough fat reserves for winter hibernation [13]. Many nocturnally migrating insects, such as migratory Lepidoptera in the family Noctuidae, are pest species on a variety of crop plants [14,15]. Therefore, these predator–prey interactions are important for pest control, as is the case with *T. brasiliensis* bats in the USA who are thought to feed on approximately four billion migratory moths per night [16]. Overall, in the USA, pest suppression by bat predation may save up to $53 billion annually due to reduced pesticide costs [17].

Co-migrant interactions often occur at migratory hotspots created by topographical conditions. In Europe, one such migratory hotspot is that of the Pass of Bujaruelo, a high-altitude pass through the Pyrenees Mountains. This site has long been known to host day-flying co-migrants in the autumn as the animals migrate south for winter [18]. The first records of co-migration occurred in 1950 when ornithologists David and Elizabeth Lack recorded birds and insects migrating diurnally through the pass in large numbers [18]. Follow-up research between 2018 and 2021 revealed that huge numbers of diurnally migrating insects move through the pass each autumn following the Western European flyway [19].

Here, we examine nocturnal migration through the Pass of Bujaruelo using moth traps and bat detectors. Given the huge numbers of diurnal insects migrating through this region, and the interactions between bats and migrant insects seen in other studies, we predict that (i) nocturnal migratory insects would also use this route in large numbers; (ii) this transient food source would be used by both migrant and resident bats; and (iii) nocturnal weather conditions would influence the abundance of bat and insect migrants.

## 2. Methods

### 2.1. Location

Our study took place in the Pyrenean migration hotspot of the Pass of Bujaruelo (2273 m in elevation and 30 m in width). Although autumnal preferred migratory direction is towards the south, the pass is orientated southwest/northeast. Despite this, migration still occurs through the pass due to the channelling effect of the steep-sided Pyrenean valley, forcing the migrants through the pass under certain weather conditions [19]. We documented the nocturnal migration of insects for three consecutive autumns (September–October) in the years 2019–2021 while bat migration was studied during September 2019. The field site contains high-quality habitat, contains no artificial light sources other than our moth trap, and is located in the protected areas of the Parc national des Pyrénées in France and in Spanish region of Aragon. Permission to conduct experiments was obtained from the Parc national des Pyrénées (France, authorization numbers: 2019-67, 2020-146 and 2021-33) and the Gobierno de Aragon (Spain, authorization numbers: 500201/24/2019/02174, 500201/24/2020/01724 and 500201/24/2021/01722).

### 2.2. Nocturnal insect identification

**Figure 1.**
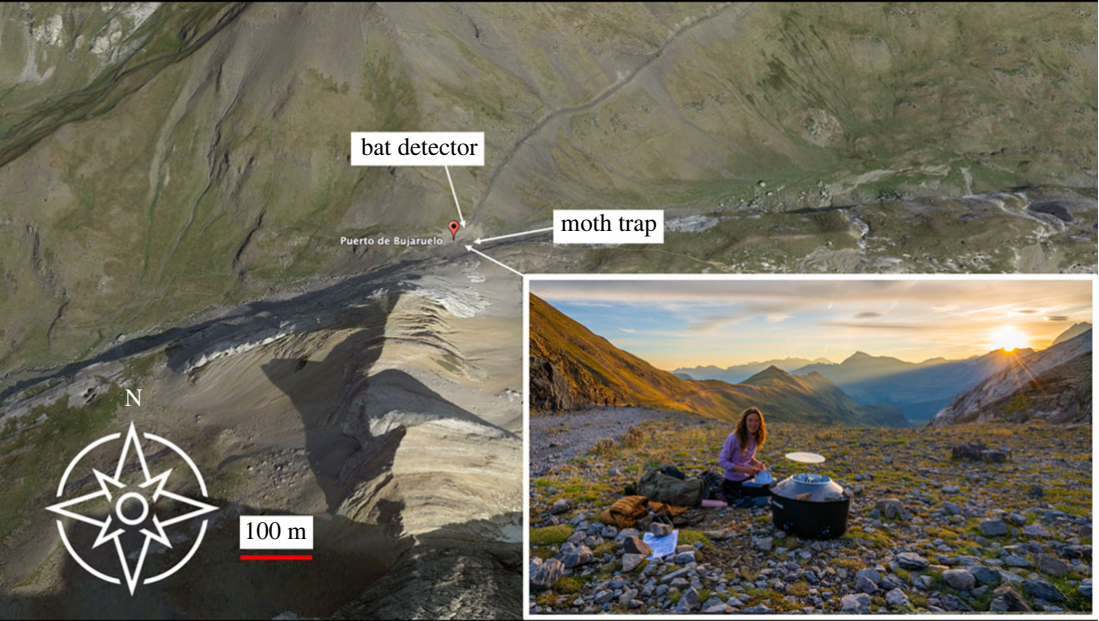
Image of the study site and the moth trap (inset) at the Pass of Bujaruelo. Location of the Anabat bat detector and the Robinson moth trap are labelled. Inset photograph ©Will Leo Hawkes.

To capture the insects migrating south during the autumns of 2019–2021, we used a Robinson moth trap complete with a LepiLED bulb, powered by a 20 000 mAhr battery pack. The moth trap was placed in the same location each season, on the northeast edge of the pass, the light shining east-north-east down the valley towards the Cirque du Gavarnie (figure 1). Each morning at approximately 09.30, moth species caught in the trap were identified to species level. The cold temperature at the pass meant that the insects were not active at collection time, suggesting that few, if any, insects were missed. Other non-moth taxa were identified to at least family level. Numbers of each species were also recorded every night. Given the harsh environmental conditions present at the location and the lack of resources available to the insects, all species were considered to show at least some migratory behaviour. Sampling occurred every night unless adverse conditions such as dangerous storms or heavy snowfall prevented access to the site.

### 2.3. Bat detector methods

The bat activity data were collected using an Anabat Express Passive Bat Detector fitted with an omnidirectional microphone. This device records at 5–250 kHz in a zero-crossing format (capturing the loudest frequency at any given moment). The detector was placed at the east opening of the pass, approximately 30 m from the moth trap—greater than the 20 m minimum advised to prevent impacts of the moth trap on bat activity [20] (figure 1)—at an angle of approximately 45° for optimum detection performance and recorded continuously from sunset to sunrise every night for the entire duration of the study, excluding adverse conditions. Variation in the frequencies of the bat calls between species affect how they can be detected. For example, the distance at which the feeding buzzes can be detected ranges from 5 m to 30 m, with *Tadarida teniotis* bats detected furthest from the Anabat detector [21]. This limitation suggests that more bats than were detected would have been present in the pass.

Bat recordings were imported into Anabat Insight by Titley Scientific (version 1.9.0). All recordings were run through a filtering system, separated by the genus/species specific metrics of their calls. Only four species/groupings were used in the analysis due to data insufficiency. This form of filtering only allows the addition of ‘species tags' to recordings which may contain any number of species. To combat this, the raw filtered data were extracted to Excel and imported into RStudio and were filtered once again using the same call metrics, on a per pulse basis as opposed to a full recording [22]. This created a dataset of bat pulse counts which will be known as ‘bat activity' in this study. Bat activity, although not the same, can be viewed as a proxy for bat occurrence in this study. Further specific bat detector methods can be found in the electronic supplementary material, file.

### 2.4. Meteorological data and analysis of environmental factors

Nocturnal weather conditions including windspeed, wind direction, temperature, cloud cover and total precipitation were obtained from meteoblue weather simulations and recorded between 19.00 and 07.00 [23]. To test which factors affected insect numbers the most, a generalized linear model (GLM) was fitted with a log link function and Poisson family [24]. The response variable was the insect counts and the explanatory variables were (i) average nightly temperature, (ii) average nightly windspeed and (iii) total nightly precipitation. The significance of the explanatory variables was determined by excluding the variable of interest before comparing the models with and without the variable using a log-likelihood test. We used corrected Akaike information criteria (AICc) model selection to evaluate which model explains the greatest amount of variation in our data using the fewest possible explanatory variables. The models were ranked to distinguish among the explanatory variables: average nightly temperature, average nightly windspeed, total nightly precipitation, a combination of the above, and their relationships to the numbers of nocturnally migrating insects. Model selection and comparison were carried out using the MuMIn and the AICcmodavg packages in R [22,25,26]. How the wind direction correlated to insect counts was analysed using a Rayleigh test of uniformity to give a mean direction, resultant length (*r*-value) and an associated *p*-value. A GLM was fitted with a log link function and quasi-Poisson family to test the factors affecting the bat numbers. The explanatory variables were the same as the nocturnal insect analysis with the addition of nocturnal insect counts as an explanatory variable.

## 3. Results

### 3.1. Nocturnal insects

**Figure 2.**
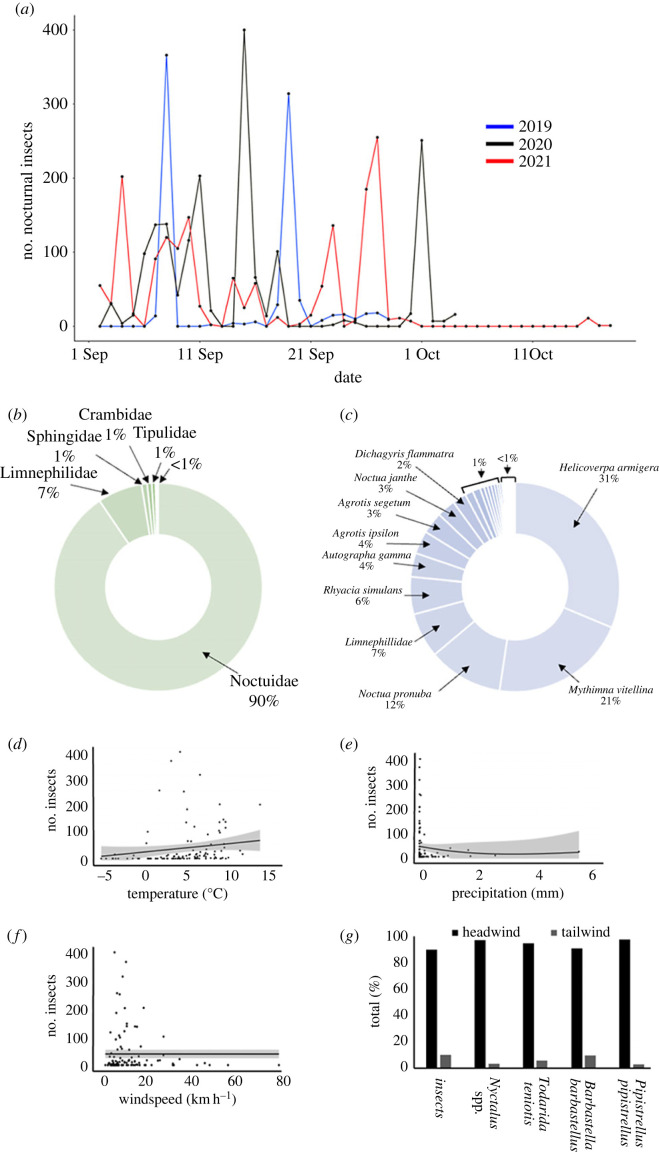
(*a*) Number of insects caught per night across three years. Average proportions of nocturnal insects showing migratory behaviour collected in the moth trap over 3 years sorted by (*b*) family and (*c*) species. For a full species list, see electronic supplementary material, tables S1 and S2. (*d*–*f*) The significant relationships between the number of insects migrating through the Pass of Bujaruelo and meteorological variables (*d*) average nightly temperature, (*e*) total nightly precipitation and (*f*) average nightly windspeed. Shaded areas represent 95% confidence intervals, and the results are based on a generalized linear model with a Poisson distribution. (*g*) The percentage of taxa recorded in a headwind or a tailwind.

A total of 4187 insects of four orders, 13 families and 66 species (or morpho-species) were collected in the Robinson moth trap over the course of the three seasons from a total of 90 trapping nights, averaging 30 nights per season. There was substantial variation between number of insects trapped per evening with a range of 0–400 and an average of 47 (s.e. ± 7.5) individuals (figure 2*a*). The Noctuidae dominated the nocturnal insects with 90% of the total assemblage (figure 2*b*). The majority (92.7%) of the insects trapped were moths (Lepidoptera), but Limnephilidae caddisflies (Trichoptera) (6.6%), Diptera (0.6%) and Hymenoptera (0.04%) were also recorded (see electronic supplementary material, table S1, for full species list). The most numerous species were *Helicoverpa armigera* (cotton bollworm, 31% of the assemblage), *Mythimna vitellina* (delicate, 21%) and *Noctua pronuba* (large yellow underwing, 12%) (figure 2*c*). For a full species list, see electronic supplementary material, tables S1 and S2.

### 3.2. The effect of environmental factors on the number of nocturnal insects migrating

To investigate the environmental factors important for migration, we analysed the influence of average nightly temperature, total nightly precipitation and average nightly windspeed on the numbers of nocturnally migrating insects. All of these explanatory variables were found to significantly affect the number of nocturnal insects caught migrating through the Pass of Bujaruelo (*p* < 0.001) (figure 2*d–f*). Temperature positively affected the nocturnal insect count number (b ± s.e. = 0.115 ± 0.004, , *p* < 0.001) while we found a negative effect of precipitation and windspeed on the number of migrating insects (b ± s.e. = −1.014 ± 0.076, $\chi_{1}^{2}=341.1$, *p* < 0.001 and b ± s.e. = 0.0051 ± 0.002, $\chi_{1}^{2}=7.3735$, *p* < 0.05 respectively). Next, we used AIC model selection to distinguish between various models describing the relationship between insect number and meteorological variables. The best-fit model, carrying 64% of the cumulative model weight, was average nightly temperature. The results of the model selection are displayed within table 1. Wind direction was found to influence the numbers of nocturnally migrating insects, with most insects caught when a headwind (wind from the southwest) was present through the Pass of Bujaruelo (figure 2*g*; Rayleigh test: 211°, *r* = 0.7783, *p* < 0.001).

**Table 1.** Model selection based on AICc for the influence of meteorological factors on the migration of nocturnal insects.

	temperature	combination of variables	precipitation	windspeed
AICc	1236.32	1239.08	1239.88	1240.22
delta AICc	0	2.77	3.56	3.9
AICc weight	0.64	0.16	0.11	0.09
cumulative weight	0.64	0.8	0.91	1

### 3.3. Bat activity in the pass

To investigate the presence of both resident and migratory bats on moth migration nights through the pass, we collected bat activity data concurrent with moth trapping during the 2019 migration season. A total of 463 511 bat call pulses were recorded with an average of 20 152 per evening and a range of 460–111 337, indicating substantial variation in activity. A total of seven species were identified (table 2) and the four species that exhibited the highest activity, the Leisler's or common noctule (*Nyctalus leisleri/noctule*) group, the European free-tailed bat (*T. teniotis*), the western barbastelle (*Barbastella barbastellus*) and the common pipistrelle (*Pipistrellus pipistrellus*) were analysed further. *Nyctalus* spp*.* activity was 2.5× higher than the next most common group of bats (*T. teniotis*).

**Table 2.** Bat activity and correlates. ‘Call pulse number' denotes a count of individual bat call pulses. *p*-values are derived from a GLM. +ve/–ve refers to ‘positive' or ‘negative' effect on species activity.

species	call pulse number	insect count (*p*)	wind direction (*p*)	windspeed (*p*)	precipitation (*p*)	temperature (*p*)
lesser or common noctule: *Nyctalus leisleri/noctule*	204 473	<0.0005 (+ve)	<0.005	0.716	0.322	0.348
European free-tailed bat: *Tadarida teniotis*	82 144	<0.0005 (+ve)	<0.005	0.6463	0.9187	0.8739
western barbastelle: *Barbastella barbastellus*	24 544	0.999	<0.05	0.7297	0.1902	0.6833
common pipistrelle: *Pipistrellus pipistrellus*	2571	0.6419	<0.05	<0.05 (–ve)	0.9805	0.8244
Savi's pipistrelle: *Hypsugo savii*	1017	n.a.	n.a.	n.a.	n.a.	n.a.
brown long-eared bat: *Plecotus auritus*	738	n.a.	n.a.	n.a.	n.a.	n.a.
soprano pipistrelle: *Pipistrellus pygmaeus*	200	n.a.	n.a.	n.a.	n.a.	n.a.

### 3.4. Factors affecting bat activity

**Figure 3.**
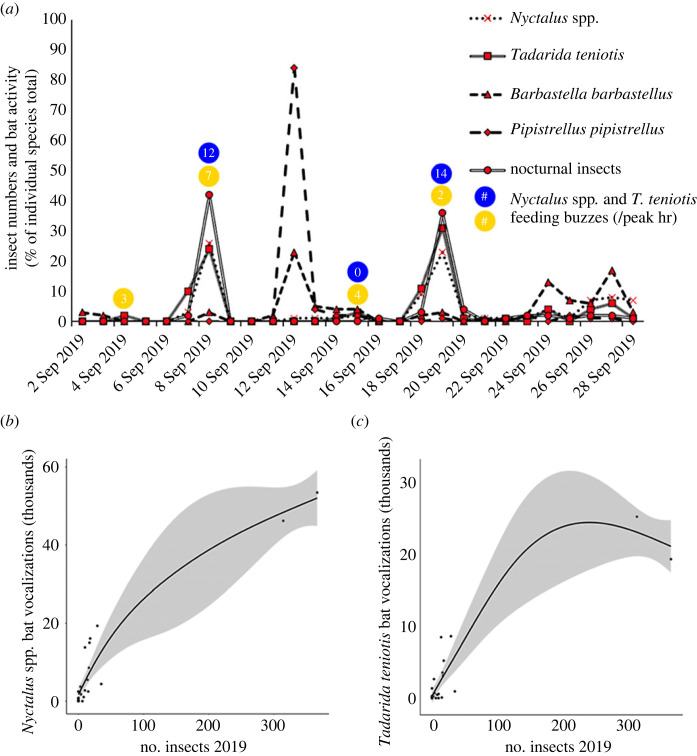
(*a*) The synchronicity in 2019 of *Nyctalus* spp., *Tadarida teniotis, Pipistrellus pipistrellus* and *Barbastella barbastellus* bat activity and nocturnal insect numbers. The *Y*-axis scale is the total percentage of each individual group (the four bats and the nocturnal insects) across the full season. The peaks represent the daily frequency expressed as a percentage of the total number across the season. Yellow (*Tadarida teniotis*) and blue (*Nyctalus* spp.) circles represent the number of feeding buzzes during the peak hour of activity on selected dates (when the bats were present with and without the presence of nocturnal insects). Significant positive relationships between the bat activity of *Nyctalus* spp. (*b*) and *Tadarida teniotis* (*c*) with the numbers of nocturnal insects migrating through the pass. Shaded areas represent 95% confidence intervals, and the results are based on a generalized linear model with a Poisson distribution.

The *Nyctalus* spp*.* activity levels at the pass were significantly influenced by increased nocturnal insect count (GLM, *F*_21,22_ = 38.89, *p* < 0.0005) (figure 3*b*) and this remained the case after removal of two outlier peak days (GLM, *F*_19,20_ = 16.328, *p* < 0.0005). Visualization of peaks of *Nyctalus* spp*.* activity and nocturnal insect count reveal clear synchronicity (see peaks on 8 and 19 September in figure 3*a*). Feeding buzzes of *Nyctalus* spp*.* bats were only recorded when insects were present. Of the other groups, only *Tadarida* activity had significant correlation with nocturnal insect count (GLM, *F*_21,22_ = 17.919, *p* < 0.0005) (figure 3*c*), where again higher numbers of insects coincided with increased bat activity levels. Feeding buzzes of *T. teniotis* were also recorded during these peak dates (figure 3*a*) but also on days when insects were not caught, suggesting that the bats were feeding on nocturnal insects not attracted to the light from the moth trap. As with the *Nyctalus* spp. activity, even after the removal of the two outlier data points, *Tadarida* activity had significant correlation with insect count (GLM, *F*_19,20_ = 8.534, *p* < 0.05). Neither the *P. pipistrellus* nor the *Barbastella barbastellus* species were significantly correlated with nocturnal insect counts. All bat group activity was found to significantly increase when the wind was blowing from a southwesterly direction (figure 2*g* and table 2). No other meteorological conditions (temperature, precipitation and windspeed) had a significant effect on any of the bat species groups other than *P. pipistrellus* whose activity was significantly increased by lower wind speeds (GLM, *F*_21,22_ = 9.2702, *p* = 0.006). For a full breakdown of the results, see electronic supplementary material, Results: ‘Influence of meteorological conditions on bats'.

## 4. Discussion

We monitored nocturnal migration through the Pyrenees in autumn, focusing on nocturnal insects moving through the Western European flyway and bat activity measured at a pass known to concentrate diurnal insect migrants. During our observation period of three consecutive autumns (September to October) in the years 2019–2021, an average of 1396 nocturnal insects were trapped each season, comprising of four orders, 13 families and 66 species. While the majority of the common nocturnal insects caught were well-known migrants such as *H. armigera, Mythimna vitllina* and *Noctua pronuba* [27–31], the fourth most abundant insect in the study, the dotted rustic (*Rhyacia simulans*), has no previous evidence of migratory behaviour, but it has long been suspected of being a migrant to the UK [32]. Rather than a recent evolution of migration in the species, this discovery is likely due to a lack of previous research. This highlights the need for continued monitoring of nocturnal insects at migratory hotspots to obtain a complete census of migratory animals and to understand their ecological roles.

During 2019, bat activity was also monitored and eight species were recorded, with the four most active being the *Nyctalus leisler/noctula* species group, the European free-tailed bat *T. teniotis, P. pipistrellus* and *B. barbastellus*. Of the four species groups analysed, only the *Nyctalus* spp*.* and *P. pipistrellus* are known to be migratory [2,33–35]. Unfortunately, due to COVID constraints on personnel, no data from subsequent years were collected. Despite this, a close relationship was found between the temporal distributions of activity for *Nyctalus* spp*.* and *T. teniotis* bats with peaks in the number of nocturnal insects. When removing the high-leverage data points from the analysis, the relationship between these bat species and the number of nocturnal insects remained significant. While the co-occurrence of bats and moths strongly suggests interaction, particularly as peaks of migrating insects coincided with increased numbers of feeding buzzes, we were not able to collect direct evidence, for example from faecal samples. By contrast, neither *P. pipistrellus* nor *B. barbastellus* activity synchronized with migratory movements of moths.

### 4.1. Environmental factors affecting insect numbers and bat activity

Numbers of nocturnal insects caught in the trap were largest when a southwesterly wind was present creating a headwind that prevented the insects from flying over the high peaks, when night-time temperatures were warmer, and when levels of precipitation were low or none. Interestingly, these meteorological conditions are broadly similar to the conditions needed for diurnal migration of other migratory species to occur through the pass, except of course for the presence of sunlight [19]. Like the nocturnal insects, the bats tended to show more activity when a southwesterly wind was present. Other than windspeed weakly affecting *P. pipistrellus* activity (a questionable result due to the leverage the peak *P. pipistrellus* day has over the model, and that wind speed has been previously seen to have no effect on this species' foraging activity [36,37]), no other meteorological conditions appeared to affect bat activity. This suggests they are less reliant on fair flying conditions than the nocturnal insects, a finding which is repeated in other studies [38–41].

*Nyctalus* spp*.* and *T. teniotis* bat activities were positively correlated to insect numbers. However, the major activity peak of *P. pipistrellus* and *B. barbastellus* occurred during a period when no migratory insects were caught. As a migratory species, *P. pipistrellus* may be simply migrating through the pass, uninfluenced by the insect migrants but possibly influenced by the lower windspeeds. By contrast, *B. barbastellus* bats are non-migratory and may be using the pass for a different reason. *B. barbastellus* bats near exclusively forage just above a forest's canopy [42,43] and there are forested areas within 4 km of either side of the pass, well within the foraging range of this species. This suggests *B. barbastellus* may be using the pass to traverse between forest habitat or to search for hibernacula [42,43]. The reason for the synchronicity of the activity peak recorded between these two species is either entirely coincidental or geared by an unknown influencer.

### 4.2. Interactions between the co-migrants

We show that there is strong synchronicity between the numbers of nocturnally migrating insects and the activity of migratory *Nyctalus* spp*.* bats (figure 3). Bat feeding buzzes recorded during these peaks of activity suggest that the bats are interacting with the insects in terms of predation. It is unclear if the Pass of Bujaruelo represents a stopover site for the *Nyctalus* spp*.*, or if the bats are feeding as they migrate. This has been termed as fly-and-forage behaviour [7,44] where a migratory individual sequesters the required nutrition by feeding on the wing [7,45]. Although it is worth noting that the interaction detailed here is more complex, as both predator and prey are migrating. Migratory moths being forced into the pass by the wind may lead to migrating individuals of *Nyctalus* to follow suit and take advantage of the abundant food source. *N. noctula* has been recorded flying at 6.7–18.6 m s^–1^ during migration and often fly into headwinds during their journey [38,39,46]. The windspeeds on peak nights were 2.5–2.8 m s^−1^, strong enough to force the nocturnal insects into the pass but not strong enough to affect the bats' migration [38,39,47]. Therefore, the bats may well be following the insects into the pass to exploit the energy rich resource. If this is the case, it is to the best of our knowledge the first recorded instance of migratory bats feeding on migratory insects while both taxa are migrating. This transient food supply provided by the nocturnally migrating insects may be an important energy source for these long-distance bat migrants [4,10,11].

Little is known regarding stopover behaviour in the *Nyctalus* genus; however, *N. noctula* and other bat species have been observed to stopover on their migrations, to refuel or enter torpor [38,39,48–50]. We note an abundance of potential sites for tree cavities, their preferred roosting site, available at lower altitudes nearby [51,52]. Alternatively, stopover behaviour can be caused by adverse conditions as well as abundant resources [53]. In September 2019, adverse weather (including snow) at the beginning of the month transitioned to calmer conditions with higher moth abundance and could have led to a stopover period and then feeding at the pass. It is difficult to separate the two theories with the type of data collected and due to the often-high variation in bat migration behaviour [38,39]. An isotopic study has shown that *N. noctula* individuals across Europe have high site fidelity towards their hibernacula year after year, but also that they were highly consistent in their migratory behaviour: 86% of bats studied were either consistently arriving from a great distance away, or they consistently stayed local to their hibernaculum [54]. This consistency suggests that the Pass of Bujaruelo could be a regularly used source of food during the autumn migration period and may be specifically targeted by the bats each year in a similar way bird migrants target known feeding stopovers [55].

*Tadarida teniotis* is thought to be a resident bat in the Pyrenees yet it too shows strong synchronicity with the numbers of nocturnally migrating insects. *Tadarida teniotis* is known to feed extensively on migratory noctuid moths during the autumn months in Portugal with Lepidoptera found in 98.6% of guano samples [13]. *Tadarida teniotis* bats give birth to their pups in the autumn [56], meaning that the arrival of nocturnal migratory insects to their home range each year could be a highly important influx of energy to help the adults and their pups develop fat stores for the winter hibernation period ahead. Similarly, Brazilian free-tailed bats (*T. brasiliensis*) in America are thought to rely on the seasonal pulse of southward migrating moths as a form of sustenance while other prey is scarce, to build up reserves prior to the bats' own migration [4,10,11]. It is worth noting that *T. teniotis* may more generally be using this influx of migratory insects by using other passes and even feeding when insects are not forced into these passes by strong headwinds. This may be why feeding buzzes are recorded on nights of low moth numbers, where the detectors are still registering bat calls from above and around the pass. This species has previously been shown to use topography, for example through orographic lift, in order to fly at high altitudes, while also reaching maximum sustained self-powered airspeeds of 135 km h^−1^ [57]. Therefore, predating on migratory insects during evenings of tailwinds is well within the bats' capabilities.

### 4.3. Ecological roles of the nocturnal migrants

The insects caught migrating nocturnally played a range of ecological roles including as pests (79%), pollinators (25%) and in nutrient transfer (100%). The most numerous species comprising 31% of the assemblage was the cotton bollworm (*H. armigera*). This species is highly polyphagous as a larva and is a major pest of crops, as are many other of the commonly recorded nocturnal insects in this study [58]. Studies have shown high levels of pesticide resistance in this species [59], and spread of this resistance may be aided by its migratory behaviour as is the case with the diamondback moth (*Plutella xylostella*). This moth is expected to expand its range northwards and increase its populations due to climate change [60] so research into the climatic drivers of its migration could prove to have important implications for understanding and preventing its spread.

Predation of these pest species by the *Nyctalus* spp*.* and *T. teniotis* bats contributes to pest control. Given the energetic requirements of bat populations, huge numbers of migratory insects are likely eaten each night, and their fat-laden bodies may aid energetically expensive long-distance migrations by the bats [61]. An American study estimated that approximately 100 million free-tailed bats (*Tadarida* sp*.*) can eat four billion *Helicoverpa zea*-sized insects (very similar to the most abundant species in our study, *H. armigera*) every night [16]. Therefore, the bats inhabiting the Pyrenees likely play a highly important role in controlling the nocturnally migrating insect pest species, thus reducing the impacts the insects have on crops at their destinations.

## 5. Conclusion

Our 3-year study of nocturnal insects migrating through a Pyrenean mountain pass has revealed a remarkable diversity of taxa, including many important pest species, following the Western European flyway south in the autumn and provides an important baseline for future comparisons. The relationship between the nocturnal insect migrants and the resident and migratory bat species described here may have important impacts for both the animals and the surrounding ecosystems. Human activities, such as habitat destruction and climate change, light pollution and the presence of wind turbines, have the potential to disrupt these interactions [54,62–66]. As huge numbers of migrants move through the Pass of Bujaruelo each year, negative impacts from human activities at this location could be especially detrimental to the migrants. However, the pass is well protected by the Parc national des Pyrénées meaning the risk to migration at this site is low. Long-term concurrent monitoring of bats and nocturnal insects in the pass could be highly productive in understanding these threats, the interactions between these species groups, and could be supplemented by faecal sampling of the bats to elucidate their dietary preferences. Future studies will help to understand, protect and preserve these animals to ensure the continuation of the vital roles they play.

## Data Availability

The data are provided in electronic supplementary material [67].

## References

[1] Chapman JW, Bell JR, Burgin LE, Reynolds DR, Pettersson LB, Hill JK, Bonsall MB, Thomas JA. 2012 Seasonal migration to high latitudes results in major reproductive benefits in an insect. Proc. Natl Acad. Sci. USA **109**, 14 924-14 929. (10.1073/pnas.1207255109) PMC3443120 22927392

[2] Dondini G, Rutkowski T, Vergari S, Wojtaszyn G. 2012 Long distance migration of female Leisler's bat (*Nyctalus leisleri*) from Italy to Poland. Hystrix **23**, 95.

[3] Dingle H. 2014 Migration: the biology of life on the move. Oxford, UK: Oxford University Press.

[4] Cohen EB, Satterfield DA. 2020 ‘Chancing on a spectacle’: co-occurring animal migrations and interspecific interactions. Ecography **43**, 1657-1671. (10.1111/ecog.04958)

[5] Alerstam T, Hedenström A, Åkesson S. 2003 Long-distance migration: evolution and determinants. Oikos **103**, 247-260. (10.1034/j.1600-0706.2003.12559.x)

[6] Moore FR. 2018 Biology of landbird migrants: a stopover perspective. Wilson J. Ornithol. **130**, 1-12. (10.1676/1559-4491-130.1.1)

[7] Šuba J, Petersons G, Rydell J. 2012 Fly-and-forage strategy in the bat *Pipistrellus nathusii* during autumn migration. Acta Chiropterologica **14**, 379-385. (10.3161/150811012X661693)

[8] Krüger F, Clare EL, Symondson WOC, Keišs O, Pētersons G. 2014 Diet of the insectivorous bat *Pipistrellus nathusii* during autumn migration and summer residence. Mol. Ecol. **23**, 3672-3683. (10.1111/mec.12547) 24118366

[9] Bartonička T, Miketová N, Hulva P. 2019 High throughput bioacoustic monitoring and phenology of the greater noctule bat (*Nyctalus lasiopterus*) compared to other migratory species. Acta Chiropterologica **21**, 75-85. (10.3161/15081109ACC2019.21.1.006)

[10] Krauel JJ, Westbrook JK, McCracken GF. 2015 Weather-driven dynamics in a dual-migrant system: moths and bats. J. Anim. Ecol. **84**, 604-614. (10.1111/1365-2656.12327) 25492132

[11] Krauel JJ, Ratcliffe JM, Westbrook JK, McCracken GF. 2018 Brazilian free-tailed bats (*Tadarida brasiliensis*) adjust foraging behaviour in response to migratory moths. Can. J. Zool. **96**, 513-520. (10.1139/cjz-2017-0284)

[12] Krauel JJ, Brown VA, Westbrook JK, McCracken GF. 2018 Predator–prey interaction reveals local effects of high-altitude insect migration. Oecologia **186**, 49-58. (10.1007/s00442-017-3995-0) 29101468

[13] Mata VA, Amorim F, Corley MFV, McCracken GF, Rebelo H, Beja P. 2016 Female dietary bias towards large migratory moths in the European free-tailed bat (*Tadarida teniotis*). Biol. Lett. **12**, 20150988. (10.1098/rsbl.2015.0988) 27009885 PMC4843218

[14] Oliver AD. 1981 *Biology and illustrated key for the identification of twenty species of economically important noctuid pests*. LSU Agricultural Experiment Station Reports, no. 260.

[15] Drake VA, Drake VA, Gatehouse AG. 1995 Insect migration: tracking resources through space and time. Cambridge, UK: Cambridge University Press.

[16] Kunz TH, Whitaker JO, Wadanoli MD. 1995 Dietary energetics of the insectivorous Mexican free-tailed bat (*Tadarida brasiliensis*) during pregnancy and lactation. Oecologia **101**, 407-415. (10.1007/BF00329419) 28306955

[17] Boyles JG, Cryan PM, McCracken GF, Kunz TH. 2011 Economic importance of bats in agriculture. Science **332**, 41-42. (10.1126/science.1201366) 21454775

[18] Lack D, Lack E. 1951 Migration of insects and birds through a Pyrenean pass. J. Anim. Ecol. **1951**, 63-67.

[19] Hawkes WL et al. 2023 The most remarkable migrants—systematic analysis of the Western European insect flyway at a Pyrenean mountain pass. *BioRxiv*. (10.1101/2023.07.17.549321)

[20] Froidevaux JSP, Fialas PC, Jones G. 2018 Catching insects while recording bats: impacts of light trapping on acoustic sampling. Remote Sens. Ecol. Conserv. **4**, 240-247. (10.1002/rse2.71)

[21] Monadjem A, Shapiro JT, Mtsetfwa F, Reside AE, McCleery RA. 2017 Acoustic call library and detection distances for bats of Swaziland. Acta Chiropterologica **19**, 175-187. (10.3161/15081109ACC2017.19.1.014)

[22] R Core Team. 2022 R: a language and environment for statistical computing. Vienna, Austria: R Foundation for Statistical Computing. See https://www.R-project.org/.

[23] MeteoBlue. 2022 *MeteoBlue Weather History*. See https://www.meteoblue.com/en/historyplus (accessed June 2022).

[24] Nelder JA, Wedderburn RWM. 1972 Generalized linear models. J. R. Stat. Soc. A (General) **135**, 370-384. (10.2307/2344614)

[25] Bartoń K. 2023 MuMIn: multi-model inference*.* R package version 1.47.5. See https://CRAN.R-project.org/package=MuMIn.

[26] Mazerolle MJ. 2023 _AICcmodavg: model selection and multimodel inference based on (Q)AIC(c). R package version 2.3.2. See https://cran.r-project.org/package=AICcmodavg.

[27] Liu Y, Fu X, Feng H, Liu Z, Wu K. 2015 Trans-regional migration of *Agrotis ipsilon* (Lepidoptera: Noctuidae) in north-east Asia. Ann. Entomol. Soc. Am. **108**, 519-527. (10.1093/aesa/sav050)

[28] Jones CM, Parry H, Tay WT, Reynolds DR, Chapman JW. 2019 Movement ecology of pest *Helicoverpa*: implications for ongoing spread. Annu. Rev. Entomol. **64**, 277-295. (10.1146/annurev-ento-011118-111959) 30296859

[29] Sparks TH, Dennis RLH, Croxton PJ, Cade M. 2007 Increased migration of Lepidoptera linked to climate change. Eur. J. Entomol. **104**, 139-143. (10.14411/eje.2007.019)

[30] Guo J, Fu X, Wu X, Zhao X, Wu K. 2015 Annual migration of *Agrotis segetum* (Lepidoptera: Noctuidae): observed on a small isolated island in northern China. PLoS ONE **10**, e0131639. (10.1371/journal.pone.0131639) 26114576 PMC4482664

[31] Chapman JW, Nesbit RL, Burgin LE, Reynolds DR, Smith AD, Middleton DR, Hill JK. 2010 Flight orientation behaviors promote optimal migration trajectories in high-flying insects. Science **327**, 682-685. (10.1126/science.1182990) 20133570

[32] Skinner B, Wilson D. 1997 Moths of the British Isles, 2nd edn. Harmondsworth, UK: Viking.

[33] Kaňuch P, Krištín A, Krištofík J. 2005 Phenology, diet, and ectoparasites of Leisler's bat (*Nyctalus leisleri*) in the Western Carpathians (Slovakia). Acta Chiropterologica **7**, 249-257. (10.3161/1733-5329(2005)7[249:PDAEOL]2.0.CO;2)

[34] Jones G. 1995 Flight performance, echolocation and foraging behaviour in noctule bats *Nyctalus noctula*. J. Zool. **237**, 303-312. (10.1111/j.1469-7998.1995.tb02764.x)

[35] Kaňuch P, Janečková K, Krištín A. 2005 Winter diet of the noctule bat *Nyctalus noctula*. Folia Zool. **54**, 53-60.

[36] Maier C. 1992 Activity patterns of pipistrelle bats (*Pipistrellus pipistrellus*) in Oxfordshire. J. Zool. **228**, 69-80. (10.1111/j.1469-7998.1992.tb04433.x)

[37] Swift SM. 1980 Activity patterns of pipistrelle bats (*Pipistrellus pipistrellus*) in north-east Scotland. J. Zool. **190**, 285-295. (10.1111/j.1469-7998.1980.tb01428.x)

[38] O'Mara MT, Wikelski M, Kranstauber B, Dechmann DKN. 2019 First three-dimensional tracks of bat migration reveal large amounts of individual behavioral flexibility. Ecology **100**, e02762. (10.1002/ecy.2762) 31127630

[39] O'Mara MT, Scharf AK, Fahr J, Abedi-Lartey M, Wikelski M, Dechmann DKN, Safi K. 2019 Overall dynamic body acceleration in straw-colored fruit bats increases in headwinds but not with airspeed. Front. Ecol. Evol. **7**, 200. (10.3389/fevo.2019.00200)

[40] Widerin K, Reiter G. 2017 Bat activity at high altitudes in the Central Alps, Europe. Acta Chiropterologica **19**, 379-387. (10.3161/15081109ACC2017.19.2.014)

[41] Voigt CC, Schneeberger K, Voigt-Heucke SL, Lewanzik D. 2011 Rain increases the energy cost of bat flight. Biol. Lett. **7**, 793-795. (10.1098/rsbl.2011.0313) 21543394 PMC3169076

[42] Hillen J, Kiefer A, Veith M. 2009 Foraging site fidelity shapes the spatial organisation of a population of female western barbastelle bats. Biol. Conserv. **142**, 817-823. (10.1016/j.biocon.2008.12.017)

[43] Sierro A, Arlettaz R. 1997 Barbastelle bats (Barbastella spp.) specialize in the predation of moths: implications for foraging tactics and conservation. Acta Oecologica **18**, 91-106. (10.1016/S1146-609X(97)80067-7)

[44] Cryan PM, Brown AC. 2007 Migration of bats past a remote island offers clues toward the problem of bat fatalities at wind turbines. Biol. Conserv. **139**, 1-11. (10.1016/j.biocon.2007.05.019)

[45] Krauel JJ, McCracken GF. 2013 Recent advances in bat migration research. In Bat evolution, ecology, and conservation (eds R Adams, S Pedersen), pp. 293-313. New York, NY: Springer. (10.1007/978-1-4614-7397-8_15)

[46] Dechmann DKN, Wikelski M, Ellis-Soto D, Safi K, O'Mara MT. 2017 Determinants of spring migration departure decision in a bat. Biol. Lett. **13**, 20170395. (10.1098/rsbl.2017.0395) 28931730 PMC5627173

[47] Brabant R, Laurent Y, Muteti J, Jonge Poerink B, Degraer S. 2019 The influence of meteorological conditions on the presence of Nathusius’ pipistrelle (*Pipistrellus nathusii*) at sea. In Environmental impacts of offshore wind farms in the Belgian part of the North Sea (eds S Degraer, R Brabant, B Rumes, L Vigin), pp. 117-124. Brussels, Belgium: Royal Belgian institute of Natural Sciences.

[48] Dechmann DKN, Wikelski M, Varga K, Yohannes E, Fiedler W, Safi K, Burkhard W-D, O'Mara MT. 2014 Tracking post-hibernation behavior and early migration does not reveal the expected sex-differences in a ‘female-migrating’ bat. PLoS ONE **9**, e114810. (10.1371/journal.pone.0114810) 25517947 PMC4269398

[49] McGuire LP, Jonasson KA, Guglielmo CG. 2014 Bats on a budget: torpor-assisted migration saves time and energy. PLoS ONE **9**, e115724. (10.1371/journal.pone.0115724) 25551615 PMC4281203

[50] McGuire LP, Guglielmo CG, Mackenzie SA, Taylor PD. 2012 Migratory stopover in the long-distance migrant silver-haired bat, *Lasionycteris noctivagans*. J. Anim. Ecol. **81**, 377-385. (10.1111/j.1365-2656.2011.01912.x) 21954938

[51] Ruczynski I, Kalko EKV, Siemers BM. 2007 The sensory basis of roost finding in a forest bat, *Nyctalus noctula*. J. Exp. Biol. **210**, 3607-3615. (10.1242/jeb.009837) 17921162

[52] Mackie IJ, Racey PA. 2007 Habitat use varies with reproductive state in noctule bats (*Nyctalus noctula*): implications for conservation. Biol. Conserv. **140**, 70-77. (10.1016/j.biocon.2007.07.031)

[53] Jonasson KA. 2017 The effects of sex, energy, and environmental conditions on the movement ecology of migratory bats. Doctoral dissertation, University of Western Ontario, Canada.

[54] Lehnert LS et al. 2018 Variability and repeatability of noctule bat migration in Central Europe: evidence for partial and differential migration. Proc. R. Soc. B **285**, 20182174. (10.1098/rspb.2018.2174) PMC6304056 30963889

[55] Cohen EB, Horton KG, Marra PP, Clipp HL, Farnsworth A, Smolinsky JA, Sheldon D, Buler JJ. 2021 A place to land: spatiotemporal drivers of stopover habitat use by migrating birds. Ecol. Lett. **24**, 38-49. (10.1111/ele.13618) 33026159

[56] Dietz C, von Helversen O, Nill D, Lina PHC, Hutson AM. 2009 Bats of Britain, Europe and northwest Africa. London, UK: A. & C. Black.

[57] O'Mara MT et al. 2021 Bats use topography and nocturnal updrafts to fly high and fast. Curr. Biol. **31**, 1311-1316. (10.1016/j.cub.2020.12.042) 33545045

[58] Cunningham JP, Zalucki MP, West SA. 1999 Learning in *Helicoverpa armigera* (Lepidoptera: Noctuidae): a new look at the behaviour and control of a polyphagous pest. Bull. Entomol. Res. **89**, 201-207. (10.1017/S0007485399000310)

[59] Mironidis GK, Kapantaidaki D, Bentila M, Morou E, Savopoulou-Soultani M, Vontas J. 2013 Resurgence of the cotton bollworm *Helicoverpa armigera* in northern Greece associated with insecticide resistance. Insect Sci. **20**, 505-512. (10.1111/j.1744-7917.2012.01528.x) 23955946

[60] Huang J. 2021 Effects of climate change on different geographical populations of the cotton bollworm *Helicoverpa armigera* (Lepidoptera, Noctuidae). Ecol. Evol. **11**, 18 357-18 368. (10.1002/ece3.8426) PMC8717297 35003678

[61] Angelo MJ, Slansky Jr FS. 1984 Body building by insects: trade-offs in resource allocation with particular reference to migratory species. Florida Entomol. **67**, 22. (10.2307/3494102)

[62] Burt CS, Kelly JF, Trankina GE, Silva CL, Khalighifar A, Jenkins-Smith HC, Fox AS, Fristrup KM, Horton KG. 2023 The effects of light pollution on migratory animal behavior. Trends Ecol. Evol. **38**, 355-368. (10.1016/j.tree.2022.12.006) 36610920

[63] Boyes DH, Evans DM, Fox R, Parsons MS, Pocock MJO. 2021 Is light pollution driving moth population declines? A review of causal mechanisms across the life cycle. Insect Conserv. Divers. **14**, 167-187. (10.1111/icad.12447)

[64] Laforge A, Pauwels J, Faure B, Bas Y, Kerbiriou C, Fonderflick J, Besnard A. 2019 Reducing light pollution improves connectivity for bats in urban landscapes. Landscape Ecol. **34**, 793-809. (10.1007/s10980-019-00803-0)

[65] Baerwald EF, D'Amours GH, Klug BJ, Barclay RMR. 2008 Barotrauma is a significant cause of bat fatalities at wind turbines. Curr. Biol. **18**, R695-R696. (10.1016/j.cub.2008.06.029) 18727900

[66] Rydell J, Bach L, Dubourg-Savage M-J, Green M, Rodrigues L, Hedenström A. 2010 Mortality of bats at wind turbines links to nocturnal insect migration? Eur. J. Wildlife Res. **56**, 823-827. (10.1007/s10344-010-0444-3)

[67] Hawkes WL, Davies K, Weston S, Moyes K, Chapman JW, Wotton KR. 2023 Bat activity correlated with migratory insect bioflows in the Pyrenees. Figshare. (10.6084/m9.figshare.c.6764148) PMC10427818 37593718

